# Prediction of high nodal burden with ultrasound and magnetic resonance imaging in clinically node-negative breast cancer patients

**DOI:** 10.1186/s40644-019-0191-y

**Published:** 2019-02-01

**Authors:** Won Hwa Kim, Hye Jung Kim, So Mi Lee, Seung Hyun Cho, Kyung Min Shin, Sang Yub Lee, Jae Kwang Lim

**Affiliations:** 1 Department of Radiology, School of Medicine, Kyungpook National University, Kyungpook National University Chilgok Hospital, 807 Hoguk-ro, Buk-gu, Daegu, 41404 Republic of Korea; 2 Department of Radiology, School of Medicine, Kyungpook National University, Kyungpook National University Hospital, Daegu, South Korea

**Keywords:** Breast cancer, Axilla, Lymph nodes, Axillary nodes, Ultrasound, Magnetic resonance imaging

## Abstract

**Background:**

Although the role of axillary imaging has been redirected for predicting high nodal burden rather than predicting nodal metastases since ACOSOG Z1011 trial, it remains unclear whether and how axillary lymph node (ALN) characteristics predicts high nodal burden. Our study was aimed to evaluate the predictive value of imaging characteristics of ALNs at ultrasound and magnetic resonance imaging (MRI) for prediction of high nodal burden (≥3 metastatic ALNs) in clinically node-negative breast cancer patients.

**Methods:**

Clinicopathological and imaging characteristics were evaluated in patients with ultrasound (*n* = 312) and MRI (*n* = 256). Imaging characteristics include number of suspicious ALNs and cortical morphologic changes (grade 1, cortical thickness < 2 mm; grade 2, 2–5 mm; grade 3, ≥5 mm or fatty hilum loss). Odds ratios (ORs) were calculated using multivariate analysis.

**Results:**

For ultrasound, higher (≥2) T stage (OR = 5.65, *P* = .005), higher number of suspicious ALNs (2 suspicious ALNs, OR = 6.52, *P* = .019; ≥ 3 suspicious ALNs, OR = 21.08, *P* = .005), and grade 3 of cortical morphologic changes (OR = 9.85, *P* = .023) independently associated with high nodal burden. For MRI, higher (≥2) T stage (OR = 5.17, *P* = .011) and higher number of suspicious ALNs (2 suspicious ALNs, OR = 69.00, *P* = .001; ≥ 3 suspicious ALNs, OR = 93.55, *P* < .001) were independently associated with high nodal burden. Among patients with 2 suspicious ALNs, those with grade 3 cortical morphologic change at ultrasound had a higher rate of high nodal burden than those with grade 2 (60.0% [3/5] vs. 25.0% [2/8]).

**Conclusions:**

A higher number of suspicious ALNs is an independent predictor for high nodal burden. Further stratification can be achieved by incorporating assessment of ultrasound-based cortical morphologic changes.

## Background

The surgical evaluation of axillary lymph node (ALN) metastases is crucial for guiding further treatment of breast cancer patients. For the patients with clinically node-negative diseases, sentinel lymph node biopsy (SLNB) is the gold standard for assessing ALN metastasis, and further axillary lymph node dissection (ALND) is generally not required if metastatic ALNs were not detected in SLNB [1, 2]. Indeed, even if the metastases are observed, but are limited to 1 or 2 ALNs (i.e., low nodal burden), large studies by the American College of Surgeons Oncology Group (ACOSOG) Z1011 and International Breast Cancer Study Group (IBCSG) 23–01 trials have shown that ALND offers no additional diagnostic or therapeutic benefit [3, 4].

Since the publication of ACOSOG Z1011 trial, the role of axillary imaging has been redirected for predicting high nodal burden (≥ 3 metastatic ALNs) rather than predicting the presence of nodal metastases. Predicting high nodal burden can facilitate individualized treatment strategies for applying neoadjuvant chemotherapy and selecting type of initial axillary surgery (SLNB vs. ALND), as well as for guiding adjuvant radiotherapy. Ultrasound is generally used in evaluating nodal metastases that is easy to perform without radiation or contrast injection. Magnetic resonance imaging (MRI) can be used to evaluate nodal metastases with advantages over ultrasound for the visualization of the entire area of the axilla irrespective of patient characteristics (e.g., obesity) or the experience of breast imager. Recent studies have reappraised the role of ultrasound and MRI in predicting high nodal burden [5–9]. However, it remains unclear whether and how ALN imaging characteristics predicts high nodal burden. We also sought to determine if the cortical morphological changes of ALNs predicted high nodal burden, as well as the number of suspicious ALNs, generally used in previous studies. Thus, the purpose of this study was to evaluate the independent values of the ALN imaging characteristics at ultrasound and MRI for predicting high nodal burden in clinically node-negative breast cancer patients.

## Methods

### Ethical considerations

This study was approved by the Institutional Review Board of Kyungpook National University Chilgok Hospital. The requirement for informed consent was waived for this retrospective analysis of observational registry.

### Patients

A total of 451 breast cancer patients who had preoperative ultrasound or MRI and underwent axillary surgery for nodal staging between December 2016 and November 2017 at Kyungpook National University Chilgok Hospital were identified from an observational registry of our institution. The following cases were excluded: patients who received neoadjuvant chemotherapy (*n* = 80); patients who underwent excisional or vacuum-assisted biopsy (*n* = 42); patients with synchronous bilateral breast cancers (*n* = 13); and patients with clinically node-positive breast cancer (n = 4). Therefore, 312 patients with clinically node-negative breast cancer were included in our retrospective analysis.

### Image acquisition and analysis

Axillary ultrasound was performed by two breast radiologists (H.J.K. and W.H.K., with 18 and 10 years of experience, respectively) with iU22 system (Philips-Advanced Technology Laboratories, Bothell, WA, USA) equipped with a 50 mm L12–5 MHz transducer and Aixplorer system (SuperSonic Imagine, Aix en Provence, France) equipped with a 50 mm SL15–4 MHz transducer. Breast MRI was performed in prone position using a 3.0-T system (Discovery MR750, GE Healthcare, Waukesha, WI) with a dedicated eight-channel surface breast coil. Each patient was administered 0.1 mL/kg of gadobutrol contrast agent (Gadovist, Bayer Schering Pharma, Berlin, Germany) injected at a rate of 1 mL/s. Axial T1-weighted images (repetition time/echo time [TR/TE]: 746/10; matrix: 352 × 256; slice thickness: 3 mm) and axial fat-suppressed T2-weighted images (TR/TE, 8087/88; matrix, 384 × 256; slice thickness, 3 mm) were acquired. Dynamic contrast-enhanced bilateral axial MR examination included one precontrast and five postcontrast phases using three-dimensional gradient-echo, fat-suppressed, T1-weighted imaging (TR/TE, 4/2; matrix, 288 × 416; flip angle, 15°; slice thickness, 1 mm).

Radiologists assigned for ultrasound and MRI interpretation documented tumor size, the number of suspicious ALNs, and imaging characteristics of the most suspicious ALN in our registry database. Imaging characteristics were evaluated as follows: cortical thickness, short diameter (SD), long diameter (LD), and the presence of fatty hilum loss. For the cortical thickness, SD, and LD assessments, radiologists were required to measure using the non-enhanced, fat-suppressed T1-weighted images to avoid errors due to the hilar vascular density caused by contrast enhancement. For the determination of presence of fatty hilum loss, radiologists were required to perform a comprehensive review of all the sequences [10]. The ALN was considered to be suspicious when one or more findings were noted as follows: cortical thickness > 2 mm and presence of eccentric cortical thickening or fatty hilum loss [9, 11–14].

### Reference standard

All patients underwent SLNB or axillary sampling (AS) with or without ALND for axillary nodal staging, and the final pathological nodal status was determined based on the examination of the surgical specimens. For patients with no suspicious ALNs at axillary imaging, the surgeons generally performed SLNB or AS. AS is defined when non-SLNs are removed in addition to removal of SLNs [15]. All non-SLNs that were suspicious on inspection or palpation were removed and submitted for intraoperative frozen sections. For patients with frozen biopsy samples (SLN and non-SLNs) that revealed metastasis, ALND was generally performed for complete removal of all nodes. ALNs were examined using hematoxylin and eosin staining, and classified as negative or positive for metastases. All histopathologic evaluations were performed by two pathologists with 18 and 10 years of experience in breast pathology.

### Data collection and statistical analysis

The following clinicopathological information was evaluated: age, histologic type, pathological T stage, multifocality, tumor location, histologic grade, estrogen receptor (ER), progesterone receptor (PR), human epidermal growth factor receptor 2 (HER2) status and type of axillary surgery. The expression of ER, PR, and HER2 was assessed by immunohistochemical staining. Quantification of ER and PR expression was performed using the Allred score: a score of > 2 was considered positive [16]. Tumors expressing ER and/or PR were defined as hormone receptor (HR)-positive. A HER2 score of 0 or 1 was considered HER2-negative, a value of 3 was considered HER2-positive, and a value of 2 was considered equivocal. For equivocal cases, silver-enhanced in situ hybridization was performed, and a HER2/CEP17 ratio of ≥2 or HER2/CEP17 ratio of < 2 with an average HER2 copy number of ≥6 were considered HER2-positive [17].

The clinicopathological characteristics were compared using a chi-square test between 1) patients undergoing ultrasound vs MRI and 2) patients with low (≤ 2 metastatic ALNs) and high (≥ 3 metastatic ALNs) nodal burden. The each imaging characteristic was classified into three groups and compared between groups with low and high nodal burden using chi-square for trend. Cortical morphologic changes were classified as grade 1–3: grade 1,cortical thickness < 2 mm; grade 2, 2–5 mm; grade 3, ≥ 5 mm or the presence of fatty hilum loss. Odds ratios (ORs) and 95% confidence intervals (95% CIs) for predicting high nodal burden were calculated with univariate logistic regression analysis, and variables with *P* < .05 were selected for the final multivariate model. All statistical analyses were performed with SPSS version 24.0 (Chicago, IL, USA) and MedCalc version 17.1 (Mariakerke, Belgium). Two-tailed *P* values of < 0.05 were considered statistically significant.

## Results

Clinicopathological characteristics in 312 patients are shown in Table 1. Mean patient age was 53.0 years (range, 23.0–85.0 years). The median number of sampled ALNs was 4 (range, 1–34). All patients underwent ultrasound, and 256 patients underwent MRI prior to surgery. There were no significant differences in the clinicopathological characteristics between the ultrasound and MRI groups. Among all patients, 19 patients (6.1%) were classified as having high nodal burden, whereas 293 patients (93.9%) were classified as having low nodal burden. Among 256 patients with MRI, 17 patients (6.6%) were classified as having high nodal burden, whereas 237 patients (93.4%) were classified as having low nodal burden.

**Table 1:** Clinicopathological characteristics of patients with ultrasound and MRI

Characteristics	Patients with ultrasound (*n* = 312)	Patients with MRI (*n* = 256)	*P* value
Age			.926
≤ 50 years	156 (50.0%)	129 (50.4%)	
> 50 years	156 (50.0%)	127 (49.6%)	
Histologic type			
Invasive ductal	288 (92.3%)	236 (92.2%)	.945
Invasive lobular	12 (3.8%)	11 (4.3%)	
Others^a^	12 (3.8%)	9 (3.5%)	
Pathological T stage			.945
T1	236 (75.6%)	195 (76.2%)	
≥ T2	76 (24.4%)	61 (23.8%)	
Tumor focality			.970
Unifocal	247 (79.2%)	203 (79.3%)	
Multifocal/multicentric	65 (20.8%)	53 (20.7%)	
Tumor location			.859
Upper outer	150 (48.1%)	125 (48.8%)	
Others^b^	162 (51.9%)	131 (51.2%)	
Histologic grade			.636
Low	33 (10.6%)	24 (9.4%)	
Moderate or High	279 (89.4%)	232 (90.6%)	
HR status			.720
Negative	62 (19.9%)	54 (21.1%)	
Positive	250 (80.1%)	202 (78.9%)	
HER2 status^c^			.591
Negative	232 (80.6%)	188 (78.7%)	
Positive	56 (19.4%)	51 (21.3%)	
Type of axillary surgery			.903
SLNB	59 (18.9%)	45 (17.6%)	
AS	224 (71.8%)	188 (73.4%)	
ALND	29 (9.3%)	23 (9.0%)	

*HR* hormone receptor, *HER2* human epidermal growth factor receptor 2, *SLNB* sentinel lymph node biopsy, *AS* axillary sampling, *ALND* axillary lymph node dissection

^a^Others include mucinous cancer (*n* = 10) and metaplastic cancer (n = 2)

^b^Other include upper inner, lower inner, lower outer, and subareolar

^c^HER2 status was only available in 288 patients with ultrasound and 239 patients with MRI

Clinicopathological characteristics of patients with high and low nodal burden are demonstrated in Table 2. Higher (≥2) T stage was more frequently found in the high nodal burden group than in the low nodal burden group (15.8% vs. 3.0%, *P* < .001). Other clinicopathological characteristics were not significantly different between the two groups. Imaging characteristics of the ALNs in groups with high and low nodal burden are demonstrated in Table 3. Among patients with ultrasound, there were significant differences between two groups in the number of suspicious ALNs (*P* < .001), cortical morphologic features (*P* < .001), and SD (*P* = .009). Among patients with MRI, there were significant differences between two groups in the number of suspicious ALNs (*P* < .001), cortical morphologic features (*P* < .001), SD (*P* = .025), and LD (*P* = .036).

**Table 2:** Clinicopathological characteristics of patients with high and low nodal burden

Characteristics	Low nodal burden (*n* = 293)	High nodal burden (*n* = 19)	*P* value
Age			.478
≤ 50 years	145 (92.9%)	11 (7.1%)	
> 50 years	148 (94.9%)	8 (5.1%)	
Pathological T stage			<.001
T1	229 (97.0%)	7 (3.0%)	
≥ T2	64 (84.2%)	12 (15.8%)	
Histologic type			.220
Invasive ductal	269 (93.4%)	19 (6.6%)	
Invasive lobular	12 (100.0%)	0	
Others^a^	12 (100.0%)	0	
Tumor focality			.544
Unifocal	233 (94.3%)	14 (5.7%)	
Multifocal/multicentric	60 (92.3%)	5 (7.7%)	
Tumor location			.175
Upper outer	138 (92.0%)	12 (8.0%)	
Others^b^	155 (95.7%)	7 (4.3%)	
Histologic grade			.438
Low	32 (97.0%)	1 (3.0%)	
Moderate or High	261 (93.5%)	18 (6.5%)	
HR status			.468
Negative	57 (91.9%)	5 (8.1%)	
Positive	236 (94.4%)	14 (5.6%)	
HER2 status ^c^			.759
Negative	217 (93.5%)	15 (6.5%)	
Positive	53 (94.6%)	3 (5.4%)	

*HR* hormone receptor, *HER2* human epidermal growth factor receptor 2

^a^Others include mucinous cancer (*n* = 10) and metaplastic cancer (*n* = 2)

^b^Other include upper inner, lower inner, lower outer, and subareolar

^c^HER2 status was available only in 288 patients

**Table 3:** Imaging characteristics of the axillary lymph nodes (ALNs) in patients with high and low nodal burden

Characteristics	Low nodal burden	High nodal burden	*P* value
*Ultrasound (n = 312)*			
Number of suspicious ALNs			<.001
0–1	282 (96.9%)	9 (3.1%)	
2	8 (61.5%)	5 (38.5%)	
≥ 3	3 (37.5%)	5 (62.5%)	
Cortical morphologic changes^a^			<.001
Grade 1	192 (98.5%)	3 (1.5%)	
Grade 2	91 (91.9%)	8 (8.1%)	
Grade 3	10 (55.6%)	8 (44.4%)	
SD			.009
< 5 mm	151 (96.2%)	6 (3.8%)	
5–10 mm	137 (93.2%)	10 (6.8%)	
> 10 mm	5 (62.5%)	3 (37.5%)	
LD			.350
< 10 mm	69 (90.8%)	7 (9.2%)	
10–15 mm	121 (95.3%)	6 (4.7%)	
> 15 mm	103 (94.5%)	6 (5.5%)	
*MRI (n = 256)*			
Number of suspicious ALNs			<.001
0–1	221 (98.2%)	4 (1.8%)	
2	11 (61.1%)	7 (38.9%)	
≥ 3	7 (53.8%)	6 (46.2%)	
Cortical morphologic changes^a^			<.001
Grade 1	130 (97.7%)	3 (2.3%)	
Grade 2	84 (93.3%)	6 (6.7%)	
Grade 3	25 (75.8%)	8 (24.2%)	
SD			.025
< 5 mm	94 (95.9%)	4 (4.1%)	
5–10 mm	131 (93.6%)	9 (6.4%)	
> 10 mm	14 (77.8%)	4 (22.2%)	
LD			.036
< 10 mm	110 (96.5%)	4 (3.5%)	
10–15 mm	86 (92.5%)	7 (7.5%)	
> 15 mm	43 (87.8%)	6 (12.2%)	

*SD* short diameter, *LD* long diameter

^a^Cortical morphologic changes was classified as grade 1–3: grade 1, cortical thickness of the most suspicious ALN < 2 mm; grade 2, 2–5 mm; grade 3, ≥ 5 mm or the presence of fatty hilum loss

In multivariate analysis (Table 4), among patients with ultrasound, higher (≥2) T stage (OR = 5.65, *P* = .005), higher number of suspicious ALNs (2 suspicious ALNs, OR = 6.52, *P* = .019; ≥ 3 suspicious ALNs, OR = 21.08, *P* = .005), and higher grade (grade 3) of cortical morphologic changes (OR = 9.85, *P* = .023) were independently associated with high nodal burden. Among patients with MRI, higher (≥2) T stage (OR = 5.17, *P* = .011) and higher number of suspicious ALNs (2 suspicious ALNs, OR = 69.00, *P* = .001; ≥ 3 suspicious ALNs, OR = 93.55, *P* < .001) were independently associated with high nodal burden.

**Table 4:** Univariate and multivariate analyses for prediction of high nodal burden

Characteristics	Univariate Analysis		Multivariate Analysis	
	Odds Ratio	*P* value	Odds Ratio	*P* value
*Ultrasound*				
T stage				
T1	1.00		1.00	
≥ T2	6.13 (2.32, 16.22)	<.001	5.65 (1.71, 18.69)	.005
Number of suspicious ALNs				
0–1	1.00		1.00	
2	19.58 (5.34, 71.83)	<.001	6.52 (1.36, 31.28)	.019
≥ 3	52.22 (10.78, 252.97)	<.001	21.08 (2.57, 172.86)	.005
Cortical morphologic changes^a^				
Grade 1	1.00		1.00	
Grade 2	5.63 (1.46, 21.71)	.012	2.70 (0.60, 12.10)	.193
Grade 3	51.20 (11.76, 222.98)	<.001	9.85 (1.37, 71.00)	.023
SD				
< 5 mm	1.00		1.00	
5–10 mm	1.84 (0.65, 5.19)	.251	1.00 (0.28, 3.50)	.996
> 10 mm	15.10 (2.91, 78.44)	.001	1.00 (0.05, 18.36)	.998
*MRI*				
T stage				
T1	1.00		1.00	
≥ T2	6.13 (2.32, 16.22)	<.001	5.17 (1.46, 18.34)	.011
Number of suspicious ALNs				
0–1	1.00		1.00	
2	35.16 (8.94, 138.31)	<.001	69.00 (5.28, 901.25)	.001
≥ 3	47.36 (10.87, 206.38)	<.001	93.55 (7.89, 1108.67)	< .001
Cortical morphologic changes^a^				
Grade 1	1.00		1.00	
Grade 2	3.10 (0.75, 12.71)	.117	0.57 (0.05, 5.97)	.638
Grade 3	13.87 (3.44, 55.91)	<.001	0.44 (0.03, 6.96)	.557
SD				
< 5 mm	1.00		1.00	
5–10 mm	1.61 (0.48, 5.40)	.437	0.35 (0.04, 2.94)	.336
> 10 mm	6.71 (1.51, 29.95)	.013	0.57 (0.04, 8.71)	.687
LD				
< 10 mm	1.00		1.00	
10–15 mm	2.24 (0.63, 7.89)	.210	1.06 (0.17, 6.74)	.948
> 15 mm	3.84 (1.03, 14.27)	.045	1.39 (0.16, 12.28)	.765

*SD* short diameter, *LD* long diameter

^a^Cortical morphologic changes were classified on a scale of grade 1–3: grade 1, cortical thickness of the most suspicious ALN < 2 mm; grade 2, 2–5 mm; grade 3, ≥ 5 mm or the presence of fatty hilum loss

**Fig. 1:**
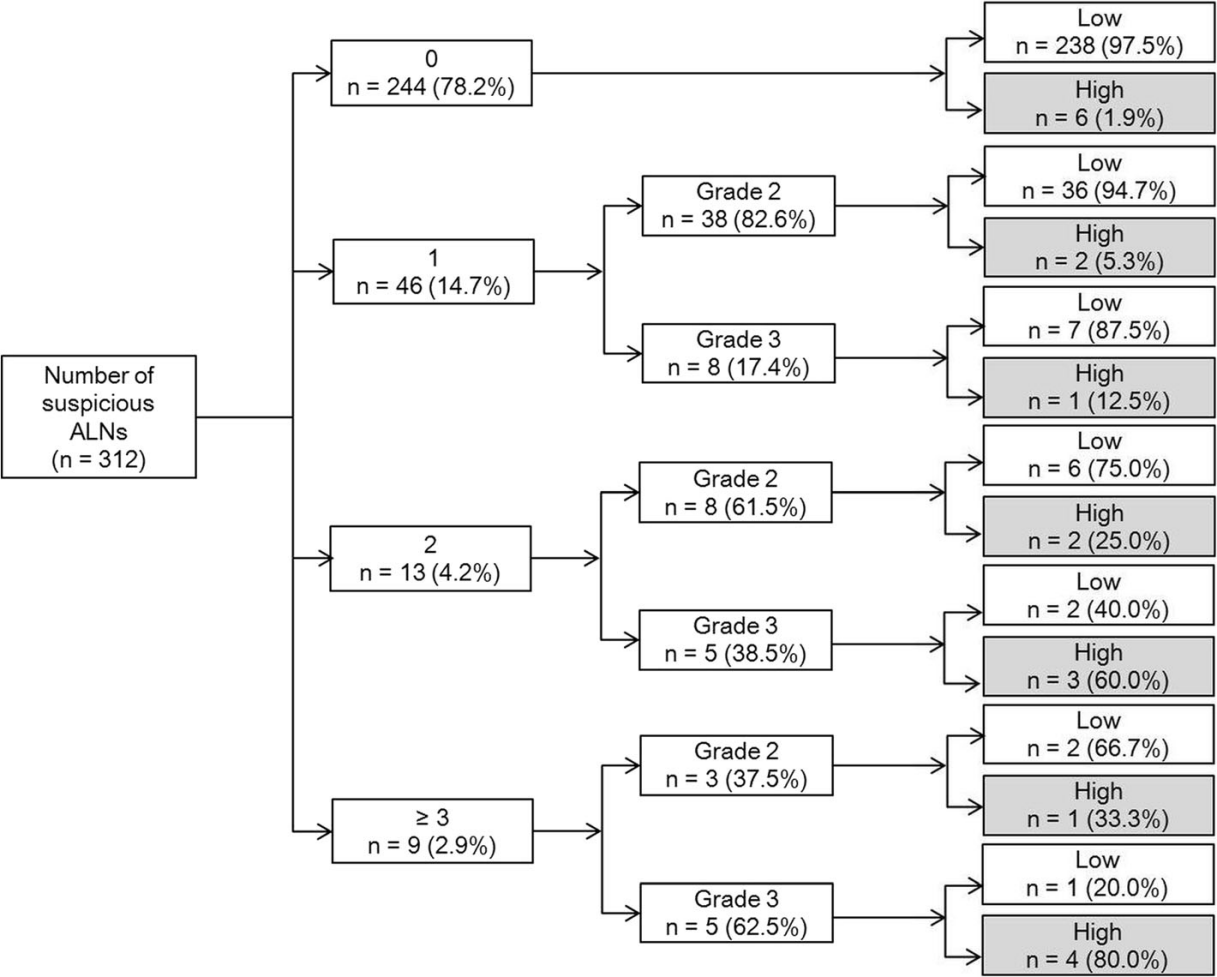
Flow chart showing the relationship among ultrasound-based number of suspicious ALNs, cortical morphologic changes, and high nodal burden

**Fig. 2:**
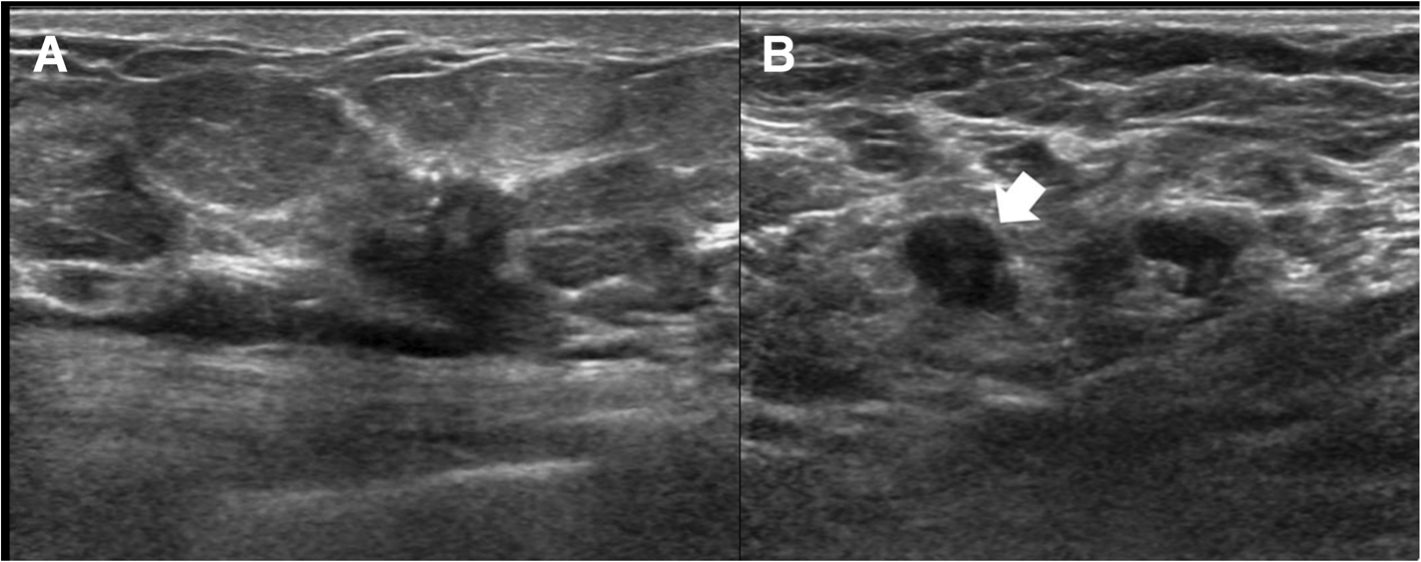
A 59-year-old woman diagnosed with invasive ductal carcinoma. **a** A confirmed, 1.2-cm malignant mass was observed in the upper inner quadrant of the left breast. **b** Two suspicious axillary lymph nodes (ALNs) were observed in the ipsilateral axilla. The most suspicious ALN (arrow) exhibits grade 3 cortical morphologic change with fatty hilum loss. This patient was confirmed to have high nodal burden, with 5 metastatic ALNs out of 18 ALNs

**Fig. 3:**
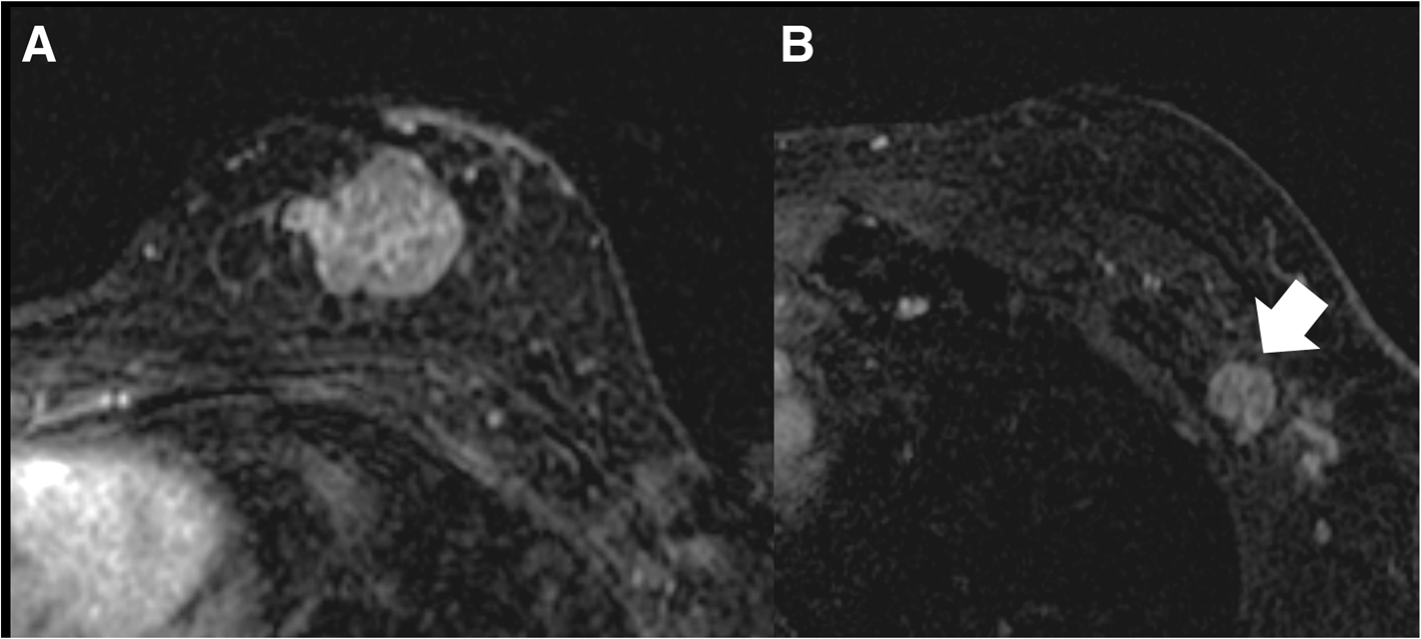
A 37-year-old woman diagnosed with invasive ductal carcinoma. **a** A confirmed, 2.3-cm malignant mass was observed in the upper inner quadrant of the left breast. **b** Two suspicious axillary lymph nodes (ALNs) were observed in the ipsilateral axilla. The most suspicious ALN (arrow) exhibits grade 3 cortical morphologic change with fatty hilum loss. This patient was confirmed to have high nodal burden, with 10 metastatic ALNs out of 10 ALNs

A flow chart showing the relationship among the number of suspicious ALN, ultrasound-based cortical morphologic changes, and high nodal burden is presented in Fig. 1. Patients with no suspicious ALN at ultrasound had a low rate of high nodal burden as 1.9% (6/244). Patients with 1 suspicious ALN also had low rates of high nodal burden as 5.3% (2/38) and 12.5% (1/8) for those with grade 2 and grade 3 cortical morphologic changes, respectively. Among patients with 2 suspicious ALNs, those with grade 3 cortical morphologic changes had higher rates of high nodal burden than those with grade 2 changes (60.0% [3/5] vs. 25.0% [2/8]) (Figs. 2 and 3). The rate of high nodal burden was highest as 80.0% (4/5) in patients with ≥3 suspicious ALNs and grade 3 morphologic changes. Among patients with MRI, the rates of high nodal burden according to the number of suspicious ALNs were 1.1% (2/181), 4.5% (2/44), 38.9% (7/18), and 46.2% (6/13) for the patients with 0, 1, 2, and ≥ 3 suspicious ALNs, respectively.

## Discussion

Preoperative knowledge of nodal burden with axillary imaging can help stratify clinically node-negative breast cancer patients into the following groups. The first group contains patients eligible for neoadjuvant chemotherapy. Although our study population only consisted of clinically node-negative patients, 20% (89/312) had metastatic ALNs and 6% (19/312) had high nodal burden, probably due to the limited accuracy of physical examination [18, 19]. By receiving neoadjuvant chemotherapy, patients with high nodal burden may experience a reduction in nodal burden, and therefore avoid ALND. The second group contains patients who would derive specific benefit from SLNB vs. ALND directly. If patients were predicted to have low nodal burden, SLNB would be important for nodal staging. In contrast, direct ALND (instead of two-staged axillary surgery) would be more effective among patients predicted to have high nodal burden. The third group contains patients who may benefit from regional nodal irradiation. According to results from the After Mapping Axillary Radiation Therapy or Surgery (AMAROS) trial, axillary radiation therapy can be used as an alternative to ALND in node-positive patients [20]. In addition, accumulating evidence suggests a potential benefit of adding regional nodal irradiation to whole-breast or post-mastectomy radiation [21, 22]. Since radiation therapy influences reconstructive surgery planning, preoperative knowledge of nodal burden would allow surgeons to develop individualized strategies.

Our study showed that patients with higher T stages and a higher number of suspicious ALNs at axillary imaging were more likely to have high nodal burden, compared to patients with lower T stages and a lower number of suspicious ALNs. Furthermore, ultrasound-based cortical morphologic change of the ALN was also an independent predictor for high nodal burden; the probability of high nodal burden increased with the increase in the grades of cortical morphologic changes. Patients with 2 suspicious ALNs and grade 3 cortical morphologic changes had a higher incidence of high nodal burden than those with 3 suspicious ALNs and grade 2 cortical morphologic changes (60.0% vs. 33.3%). Cortical morphologic change is a well-established sign of metastases as metastatic cells reside in the nodal cortex [23, 24]. Our results imply that patients with grade 3 cortical morphologic changes are more likely to have metastases in other ostensibly normal ALNs.

However, MRI-based cortical morphologic change was not independently associated with high nodal burden. There were discrepancies in predictive values of imaging characteristics predicting high nodal burden between ultrasound and MRI. This is probably due to the lower resolution of an MRI for the axilla than ultrasound; discrimination between fatty hilum and cortex may be inaccurate in an MRI with a relatively high field of view. In addition, differences in the time interval between breast biopsy and the timing of imaging acquisition (ultrasound vs. MRI) could in part account for the discrepancies in the predictive values. In our practice, ultrasound for staging was performed prior to MRI and after biopsy (usually performed in outside facilities).

Among the clinicopathological and imaging characteristics, the number of suspicious ALNs was the strongest predictor for both ultrasound and MRI. Among patients with ≥3 suspicious ALNs, the probability of having high nodal burden was 62.5% at ultrasound and 46.2% at MRI; ORs were 21.08 and 93.55 for ultrasound and MRI, respectively. Among patients with 2 suspicious ALNs, the probability of having high nodal burden was 38.5% at ultrasound and 38.9% at MRI; ORs were 6.52 and 69.00 for ultrasound and MRI, respectively. In contrast, when patients had 0–1 suspicious ALNs at preoperative imaging, the probability of having high nodal burden was very low as 3.1 and 1.8% at ultrasound and MRI, respectively. Our data are consistent with previous studies wherein detection of a limited number of suspicious ALNs at ultrasound or MRI could reliably exclude the presence of high nodal burden [6–9, 25]. However, these studies used variable criteria to exclude the presence of high nodal burden, and the definition of high nodal burden was different (pN2-N3 or ≥ 3 metastatic ALNs) among studies. Our data showed in detail how different numbers of suspicious ALNs can differently predict high nodal burden, defined as ≥3 metastatic ALNs based on ACOSOG Z1011 trial. One finding of particular interest relates to the presence of 1 or 2 suspicious ALNs at axillary imaging: detection of 1 suspicious ALN had a high negative predictive value for the presence of high nodal burden (93.5 and 95.5% for ultrasound and MRI, respectively). Even when 2 suspicious ALNs were detected at ultrasound, the probability of having high nodal burden was low as 25.0% if the ALN showed grade 2 cortical morphologic changes, but increased to 60.0% if the ALN showed grade 3 changes. Patients with latter group may require neoadjuvant chemotherapy, ALND, or adjuvant regional nodal irradiation.

This study has several limitations. First, our results should be interpreted in light of low prevalence of high nodal burden in our study population. Nevertheless, ultrasound- and MRI-based imaging characteristics were independently associated with high nodal burden, supporting the predictive value of axillary imaging. In addition, due to the limited number of patients with high nodal burden, our power to perform more stratified analyses was limited, particularly for the MRI group. Third, not all patients underwent ALND for the specific purpose of pathologic determination, and node-to-node analysis was not performed. Fourth, the role of diffusion-weighted imaging (DWI) in MRI for predicting high nodal burden was not investigated. DWI has been reported to be feasible for differentiating metastatic from non-metastatic axillary lymph node [26], and may contribute to predict high nodal burden; however, future study is needed to elucidate the role. Finally, the dedicated axillary MRI protocol using surface coil was not used in this study. Several previous studies demonstrated the role of the dedicated axillary MRI that would give rise to different criteria or predictive value from that obtained in our study [27, 28].

## Conclusions

ALN imaging characteristics at ultrasound and MRI, as well as clinicopathological characteristics can be used to predict nodal burden in clinically node-negative breast cancer patients. A higher (2 or ≥ 3) number of suspicious ALNs is an independent predictor for high nodal burden. Further stratification can be achieved by incorporating assessment of ultrasound-based cortical morphologic changes.

## Abbreviations

ACOSOG: American College of Surgeons Oncology Group
ALN: Axillary lymph node
ALND: Axillary lymph node dissection
AMAROS: After Mapping Axillary Radiation Therapy or Surgery
AS: Axillary sampling
CI: Confidence interval
ER: Estrogen receptor
HER2: Human epidermal growth factor receptor 2
HR: Hormone receptor
IBCSG: International Breast Cancer Study Group
LD: Long diameter
MRI: Magnetic resonance imaging
OR: Odds ratio
PR: Progesterone receptor
SD: Short diameter
SLNB: Sentinel lymph node biopsy
TE: Echo time
TR: Repetition time

## References

[1] Mansel RE, Fallowfield L, Kissin M, Goyal A, Newcombe RG, Dixon JM (2006). Randomized multicenter trial of sentinel node biopsy versus standard axillary treatment in operable breast cancer: the ALMANAC trial. J Natl Cancer Inst.

[2] Veronesi U, Paganelli G, Viale G, Luini A, Zurrida S, Galimberti V (2003). A randomized comparison of sentinel-node biopsy with routine axillary dissection in breast cancer. N Engl J Med.

[3] Giuliano AE, Hunt KK, Ballman KV, Beitsch PD, Whitworth PW, Blumencranz PW (2011). Axillary dissection vs no axillary dissection in women with invasive breast cancer and sentinel node metastasis: a randomized clinical trial. JAMA.

[4] Galimberti V, Cole BF, Zurrida S, Viale G, Luini A, Veronesi P (2013). Axillary dissection versus no axillary dissection in patients with sentinel-node micrometastases (IBCSG 23-01): a phase 3 randomised controlled trial. Lancet Oncol..

[5] Jackson RS, Mylander C, Rosman M, Andrade R, Sawyer K, Sanders T (2015). Normal axillary ultrasound excludes heavy nodal disease burden in patients with breast Cancer. Ann Surg Oncol.

[6] Caudle AS, Kuerer HM, Le-Petross HT, Yang W, Yi M, Bedrosian I (2014). Predicting the extent of nodal disease in early-stage breast cancer. Ann Surg Oncol.

[7] Neal CH, Daly CP, Nees AV, Helvie MA (2010). Can preoperative axillary US help exclude N2 and N3 metastatic breast cancer?. Radiology.

[8] van Nijnatten TJA, Ploumen EH, Schipper RJ, Goorts B, Andriessen EH, Vanwetswinkel S (2016). Routine use of standard breast MRI compared to axillary ultrasound for differentiating between no, limited and advanced axillary nodal disease in newly diagnosed breast cancer patients. Eur J Radiol.

[9] Hyun SJ, Kim EK, Moon HJ, Yoon JH, Kim MJ (2016). Preoperative axillary lymph node evaluation in breast cancer patients by breast magnetic resonance imaging (MRI): can breast MRI exclude advanced nodal disease?. Eur Radiol.

[10] Kim WH, Kim HJ, Lee SM, Cho SH, Shin KM, Lee SY, et al. Preoperative axillary nodal staging with ultrasound and magnetic resonance imaging: predictive values of quantitative and semantic features. Br J Radiol. 2018:20180507. 10.1259/bjr.20180507 PMC6319840 30059242

[11] Park SH, Kim EK, Park BW, Kim SI, Moon HJ, Kim MJ (2013). False negative results in axillary lymph nodes by ultrasonography and ultrasonography-guided fine-needle aspiration in patients with invasive ductal carcinoma. Ultraschall Med.

[12] You S, Kang DK, Jung YS, An YS, Jeon GS, Kim TH (2015). Evaluation of lymph node status after neoadjuvant chemotherapy in breast cancer patients: comparison of diagnostic performance of ultrasound, MRI and (1)(8)F-FDG PET/CT. Br J Radiol.

[13] Boughey JC, Ballman KV, Hunt KK, McCall LM, Mittendorf EA, Ahrendt GM (2015). Axillary ultrasound after neoadjuvant chemotherapy and its impact on sentinel lymph node surgery: results from the American College of Surgeons oncology group Z1071 trial (Alliance). J Clin Oncol.

[14] Cho N, Moon WK, Han W, Park IA, Cho J, Noh DY (2009). Preoperative sonographic classification of axillary lymph nodes in patients with breast cancer: node-to-node correlation with surgical histology and sentinel node biopsy results. AJR Am J Roentgenol.

[15] Boughey JC, Suman VJ, Mittendorf EA, Ahrendt GM, Wilke LG, Taback B (2013). Sentinel lymph node surgery after neoadjuvant chemotherapy in patients with node-positive breast cancer: the ACOSOG Z1071 (Alliance) clinical trial. JAMA.

[16] Allred DC, Harvey JM, Berardo M, Clark GM (1998). Prognostic and predictive factors in breast cancer by immunohistochemical analysis. Mod Pathol.

[17] Wolff AC, Hammond ME, Hicks DG, Dowsett M, McShane LM, Allison KH (2013). Recommendations for human epidermal growth factor receptor 2 testing in breast cancer: American Society of Clinical Oncology/College of American Pathologists clinical practice guideline update. J Clin Oncol.

[18] Sacre RA (1986). Clinical evaluation of axillar lymph nodes compared to surgical and pathological findings. Eur J Surg Oncol.

[19] Specht MC, Fey JV, Borgen PI, Cody HS 3rd (2005). Is the clinically positive axilla in breast cancer really a contraindication to sentinel lymph node biopsy?. J Am Coll Surg.

[20] Donker M, van Tienhoven G, Straver ME, Meijnen P, van de Velde CJ, Mansel RE (2014). Radiotherapy or surgery of the axilla after a positive sentinel node in breast cancer (EORTC 10981-22023 AMAROS): a randomised, multicentre, open-label, phase 3 non-inferiority trial. Lancet Oncol.

[21] Whelan TJ, Olivotto IA, Parulekar WR, Ackerman I, Chua BH, Nabid A (2015). Regional nodal irradiation in early-stage breast Cancer. N Engl J Med.

[22] Huang EH, Tucker SL, Strom EA, McNeese MD, Kuerer HM, Buzdar AU (2004). Postmastectomy radiation improves local-regional control and survival for selected patients with locally advanced breast cancer treated with neoadjuvant chemotherapy and mastectomy. J Clin Oncol.

[23] Bedi DG, Krishnamurthy R, Krishnamurthy S, Edeiken BS, Le-Petross H, Fornage BD (2008). Cortical morphologic features of axillary lymph nodes as a predictor of metastasis in breast cancer: in vitro sonographic study. AJR Am J Roentgenol.

[24] Tateishi T, Machi J, Feleppa EJ, Oishi R, Furumoto N, McCarthy LJ (1999). In vitro B-mode ultrasonographic criteria for diagnosing axillary lymph node metastasis of breast cancer. J Ultrasound Med.

[25] Moorman AM, Bourez RL, Heijmans HJ, Kouwenhoven EA (2014). Axillary ultrasonography in breast cancer patients helps in identifying patients preoperatively with limited disease of the axilla. Ann Surg Oncol.

[26] Chung J, Youk JH, Kim JA, Gweon HM, Kim EK, Ryu YH (2014). Role of diffusion-weighted MRI: predicting axillary lymph node metastases in breast cancer. Acta Radiol.

[27] Schipper RJ, Paiman ML, Beets-Tan RG, Nelemans PJ, de Vries B, Heuts EM (2015). Diagnostic performance of dedicated axillary T2- and diffusion-weighted MR imaging for nodal staging in breast Cancer. Radiology.

[28] Baltzer PA, Dietzel M, Burmeister HP, Zoubi R, Gajda M, Camara O (2011). Application of MR mammography beyond local staging: is there a potential to accurately assess axillary lymph nodes? Evaluation of an extended protocol in an initial prospective study. AJR Am J Roentgenol.

